# Impact of COVID-19 on Emergency Medicine Residency Programs: A Cross-Sectional Study in New York State

**DOI:** 10.5811/westjem.2021.10.54266

**Published:** 2022-01-18

**Authors:** Muhammad Waseem, Nidhi Garg, Bernard P. Chang, Juan Acosta, John DeAngelis, Mary E. McLean, Laura D. Melville, Timothy Pistor, Kaushal H. Shah, JoAnne Tarantelli, Susan M. Wojcik, James Gerard Ryan

**Affiliations:** * Lincoln Medical and Mental Health Center, Department of Pediatrics and Emergency Medicine, Bronx, New York; † Weill Cornell Medicine, Department of Pediatric Emergency Medicine, New York, New York; ‡ New York’s Medical College, Department of Pediatric Emergency Medicine, Valhalla, New York; § Donald and Barbara Zucker School of Medicine at Hofstra/Northwell, Hempstead, New York; ¶ South Shore University Hospital/Northwell Health, Department of Emergency Medicine, Bay Shore, New York; || Columbia University Medical Center, Department of Emergency Medicine, New York, New York; # Saint Catherine of Siena Medical Center, Department of Emergency Medicine, Smithtown, New York; ** New York Institute of Technology College of Osteopathic Medicine, Department of Emergency Medicine, Glen Head, New York; †† University of Rochester, Department of Emergency Medicine, Rochester, New York; ‡‡ St. John’s Riverside Hospital, Department of Emergency Medicine, Yonkers, New York; §§ New York Presbyterian-Brooklyn Methodist Hospital, Department of Emergency Medicine, Brooklyn, New York; ¶¶ New York Chapter American College of Emergency Physicians, Webster, New York; |||| SUNY Upstate Medical University, Department of Emergency Medicine, Syracuse, New York

## Abstract

**Introduction:**

The 2019 novel coronavirus pandemic has caused significant disruptions in the clinical operations of hospitals as well as clinical education, training, and research at academic centers. New York State was among the first and largest epicenters of the pandemic, resulting in significant disruptions across its 29 emergency medicine (EM) residency programs. We conducted a cross-sectional observational study of EM residency programs in New York State to assess the impact of the pandemic on resident education and training programs.

**Methods:**

We surveyed a cross-sectional sample of residency programs throughout New York State in June 2020, in the timeframe immediately after the state’s first “wave” of the pandemic. The survey was distributed to program leadership and elicited information on pandemic-prompted curricular modifications and other educational changes. The survey covered topics related to disruptions in medical education and sought details on solutions to educational issues encountered by programs.

**Results:**

Of the 29 accredited EM residency programs in New York State, leadership from 22 (76%) responded. Of these participating programs, 11 (50%) experienced high pandemic impact on clinical services, 21 (95%) canceled their own trainees’ off-service rotations, 22 (100%) canceled or postponed visiting medical student rotations, 22 (100%) adopted virtual conference formats (most within the first week of the pandemic wave), and 11 (50%) stopped all prospective research (excluding COVID-19 research), while most programs continued retrospective research.

**Conclusion:**

This study highlights the profound educational impact of the pandemic on residency programs in one of the hardest- and earliest-hit regions in the United States. Specifically, it highlights the ubiquity of virtual conferencing, the significant impact on research, and the concerns about canceled rotations and missed training opportunities for residents, as well as prehospital and non-physician practitioner trainees. This data should be used to prompt discussion regarding the necessity of alternate educational modalities for pandemic times and the sequelae of implementing these plans.

## INTRODUCTION

The 2019 novel coronavirus (COVID-19) pandemic has upended broad swaths of the medical and educational world. The World Health Organization declared COVID-19 a global pandemic on March 11, 2020,^1^ and on March 18, 2020, Governor Andrew Cuomo of New York State (NYS) declared a state of emergency. The disruptive effects of the pandemic influenced individuals’ lives broadly across the healthcare system. The state of New York was one of the earliest COVID-19 epicenters across the nation. It is also home to the largest number of emergency medicine (EM) residencies in the country, most of which are located within the New York City epidemic area. As the pandemic crisis evolved, the Accreditation Council for Graduate Medical Education (ACGME) monitored the needs of the graduate medical education community and provided appropriate guidance for residency training programs by declaring pandemic status for affected institutions.

These guidelines increased flexibility, allowing sponsoring programs to increase availability of physicians in clinical care settings. However, programs are required to maintain adequate resident supervision, continue meeting work hours limits, and provide alternative educational resources. Both anecdotally and through personal experiences, we learned that the pandemic resulted in canceled core rotations, conferences, other educational sessions, and research activities for EM residents in NYS. Through this study, we endeavored to document and analyze the pandemic’s disruptions on clinical training, didactic educational experiences, research programs, and wellness activities at NYS EM residency programs.

## METHODS

In this study we used a cross-sectional design, the setting was virtual, participants were offered no research incentives, and there was no funding. The institutional review board determined the study to be exempt. A survey was developed by the Research Committee of the New York chapter of the American College of Emergency Physicians (ACEP), which consists of 12 active educational faculty and research expert members representing EM residency programs in various institutional settings across NYS. The survey was developed and validated by this group using an iterative process. The questions were proposed and discussed, reworked and re-vetted until the committee reached consensus on face validity and internal consistency. The survey included program demographic questions along with questions related to pandemic-triggered academic program changes. These questions were structured to assess the overall changes to clinical schedules, lecture format, presence of external rotators, and departmental research and wellness programs. A copy of the survey is included in the Appendix.

The survey was distributed via email from the New York ACEP to program directors of the 29 ACGME-accredited EM residencies in NYS. Three contact attempts were made over the one-month period of active data collection. Only one response per program was reported, and none of the survey creators were respondents. We analyzed data using SPSS version 26 (IBM Corporation, Armonk, New York). Descriptive data was reported for each of the variables, and we used a chi square analysis to compare differences across categorical variables.

**Population Health Research Capsule**

What do we already know about this issue?

*COVID-19 significantly disrupted hospital clinical operations in New York State—the first and among the largest of the United States epicenters.*

What was the research question?

*How did the first pandemic surge impact New York State EM residency program education, training, research, and wellness?*

What was the major finding of the study?

*Programs adopted virtual conferencing, canceled clinical rotations and research, and instituted changes to wellness initiatives.*

How does this improve population health?

*Residency is crucial for training proficient physicians. Studying pandemic impacts equips educators to maintain high-quality training in the face of such disasters.*

## RESULTS

Of 29 ACGME-accredited NYS EM residencies, program directors from 22 (76%) completed the survey. Respondents self-identified their program’s primary geographical class, with 15 (68%) identifying as urban, six (27%) as suburban, and one (5%) as rural. Respondents identified their program’s primary hospital type, with 15 (68%) identifying as academic/university and seven (32%) as community. Respondents specified their residency length, with 13 (59%) indicating three-year duration and nine (41%) indicating four-year duration.

Table 1 depicts the program leadership reports of overall COVID-19 pandemic impact on off-service clinical rotations, including both aggregate and subanalyses for program length, hospital setting, and geographic location/population density. Table 2 depicts cancellation rate of the most frequently canceled off-service rotations. Notably, of all responding programs, 21 (95%) canceled at least one off-service rotation.

Table 3 depicts program leadership report rates of ACGME pandemic status placement, overall rotation cancellation rates for internal and visiting trainees, and effects on weekly conferences and research. Of the 22 participating programs, 22 (100%) reported that prior to the pandemic wave they had rotating medical students in their emergency departments, 15 (68%) had physician assistant students, 11 (50%) had emergency medical technician interns, 11 (50%) had nursing students, and 10 (45%) had nurse practitioner students. All programs did transition to virtual conferencing, ostensibly due to NYS social distancing requirements. Adoption of alternate lecture formats occurred rapidly, with 16 (73%) transitioning to virtual conference within one week of the ACGME declaring pandemic status, and 20 (91%) transitioning within four weeks. Half of programs halted all prospective research, and at least two respondents noted that only COVID-19 specific research continued.

Table 4 depicts frequency of resident wellness initiative changes. Of 22 responding programs, 20 (91%) made significant changes to resident wellness programs during this first pandemic wave.

## DISCUSSION

In the past century, there has been no opportunity other than during COVID-19 to study the effects of a worldwide pandemic on medical education and training. Understanding its impact on residency programs provides insight to better prepare teaching hospitals for future disasters, including pandemics, natural disasters, war, terrorism, electrical blackouts, etc. We found that COVID-19 impacted residency education in all participating programs in NYS, including clinical, didactic, research, and wellness experiences.

The high overall clinical impact suggests that effects were universal without regard to duration of training programs, hospital setting, or geographical location. The fact that over 90% of offsite rotations were canceled depicts significant limitations to training opportunities, amplified by the fact that rotations historically thought of as crucial to EM education were not spared. The fact that anesthesia and emergency medical services (EMS) were the most canceled rotations is a particularly powerful message. Anesthesia exposure aids in resident training on critical airway management,^2^ and EMS aids in crucial resident understanding of prehospital care (eg, EMS protocols and operations).^3^

All programs adopted virtual platforms and curricula for weekly conferences, which requires institutional investment and guidance.^4^ It has previously been suggested that the didactic portion of medical student education should transition to online curricula.^5^ Several medical schools have embraced this change, even finding ways to administer team-based exercises, interactive clinical cases, and real-time quizzes using these virtual platforms.^6^ The advantages and disadvantages of virtual didactic curricula (and the determination of whether virtual curricula can provide the same degree of knowledge acquisition as traditional in-person education) requires further study.

Over half of the participating programs reported a complete interruption of clinical research activities, and a third of programs were able to continue retrospective chart review studies. In some instances, in-person data collection was postponed due to safety considerations for research teams, while other research-related activities such as analysis and manuscript writing continued. While many non-COVID-19 research projects were paused during the pandemic, a significant increase in COVID-19 related work was noted by many participating programs. This provided novel opportunities for innovative research, including grant-funded projects, although this potentially narrowed the focus of resident institutional research. The combination of these factors may lead to significantly reduced research experiences for graduating residents.^7^

The COVID-19 pandemic produced a significant change in resident wellness programs. Survey participants reported using new methodologies to improve wellness, including soliciting or obtaining food or discounts, and virtual social gatherings. This leads us to think that on an everyday basis, procuring food during shifts can be a challenge for EM residents, presumably because there is little downtime. This knowledge suggests that such interventions are beneficial on a routine basis, reflected in the fact that many programs provide food stipends or meal cards. Beyond food incentives, participants also reported using virtual environments in creative ways (including social gatherings, group meditation, and yoga). We suspect that further innovative uses of virtual environments will be forthcoming.

## LIMITATIONS

As with any similar survey-based research, our study was potentially impacted by recall bias of participants, and this may be especially true given the physical and emotional stress experienced during the pandemic. We attempted to keep the questions as factual as possible to reduce bias that may have presented with subjective responses. However, limiting the answers to objective data may have led to oversimplification of a complex and dynamic situation. Furthermore, these questions were not from a validated source, but were crafted to extract factual answers by members of the New York ACEP Research Committee, who are experts in the field. It is unknown whether respondents perceived pressure to over- or underestimate their answers. It is also unknown how generalizable these findings are to all United States EM residency programs given that the NYS COVID-19 experience may have been unique. Despite these limitations, we believe that this study has provided useful and revealing objective data, and that this information can guide future investigations. Ultimately, more research is needed to measure the relative level of impact for residents who trained during the pandemic vs those who did not, focusing specifically on competency and milestone achievement.

## CONCLUSION

The COVID-19 pandemic has dramatically transformed education for physicians in training. It has also challenged EM residency programs to rebalance priorities of patient care, resident education, and wellness. The present study provides important information regarding the effects of COVID-19 on EM residency programs regarding these priorities. Pandemic times have called for difficult decisions to be made, for innovation, and for rapid adoption of unconventional teaching modalities, such as virtual platforms, to minimize training disruptions. They also have called for new ways to connect socially and promote wellness. Adaptability and flexibility during this challenging time is not unique to EM, but it is often recognized as one of our strongest attributes. This study highlights many of the difficult training decisions that NYS EM residency leadership made during the state’s first COVID-19 wave, and the ways in which programs have met challenges presented by the pandemic. Such discussions of the pandemic’s short-term effects on resident education are crucial, but the long-term effects of COVID-19 on EM education and training remain to be seen.

## Supplementary Information



## Figures and Tables

**Table 1:** Perceived impact of the 2019 Novel Coronavirus on off-service rotations after the first pandemic wave in New York State. Impact categories were defined by percentage of residents affected, with high, moderate, neutral, and minimal impact signifying 61–100%, 41–60%, 21–40%, and <20% of residents affected, respectively. Sub-analyses of program length, hospital setting, and geographic location revealed no significant differences in impact severity.

	Off-service rotation impact n(%)		

	High	Moderate	Minimal
Overall analysis	11(50%)	5(23%)	6(27%)
Sub- analyses			
Program length			
3-Year	6(46%)	2(16%)	5(38%)
4-Year	5(56%)	3(33%)	1(11%)
Hospital setting			
Academic	7(47%)	3(20%)	5(33%)
Community	4(57%)	2(29%)	1(14%)
Geographic location			
Rural	0(0%)	1(100%)	0(0%)
Suburban	4(25%)	1(6%)	7(69%)
Urban	7(47%)	3(20%)	5(33%)

**Table 2:** Clinical rotation cancellations. The “Other” category includes all rotations that were canceled by fewer than 10% of participating residency programs, including medical and surgical intensive care units, otorhinolaryngology, radiology, burn, coronary care unit, internal medicine, and psychiatry. EMS, emergency medical services; OB/GYN, obstetrics/gynecology.

Rotation	Cancellations n(%)
Anesthesia	14(64%)
EMS	14(64%)
Toxicology	12(55%)
OB/GYN	12(55%)
Ultrasound	9(41%)
Orthopedics	8(36%)
Research	7(32%)
Ophthalmology	5(23%)
Administration	4(18%)
Teaching	4(18%)
Trauma	3(14%)
Other	12(55%)

**Table 3:** Descriptive statistics portraying the impact of the 2019 Novel Coronavirus pandemic on hospital training environment programs in New York State, including clinical, didactic, and research experiences. Wherever applicable, percentages are calculated from total institutions reporting on the measure (programs answering “not applicable” are excluded). Also wherever applicable, the denominator is specified if fewer than 22 programs reported on a measure.

Measure	n(%)
Pandemic status placement	15(68%)
Canceled internal trainees’ off-service rotations	21(95%)
Canceled outside trainees’ visiting rotations	-
Medical students	16/22(73%)
Physician assistant students	10/15(67%)
Nurse practitioner students	7/12(58%)
Nursing students	7/11(64%)
Emergency medical technician interns	7/11(64%)
Postponed outside trainees’ visiting rotations	-
Medical students	6/22(27%)
Physician assistant students	5/15(33%)
Nurse practitioner students	5/12(42%)
Nursing students	4/11(36%)
Emergency medical technician interns	3/11(27%)
Weekly conference	-
Virtual conference format adopted	22(100%)
Limited to small groups <10	2(9%)
Changed to self-directed learning	2(9%)
Institutional research	-
Prospective research stopped	11(50%)
Prospective research continued with video/phone	6(28%)
Retrospective research stopped	1(5%)
Retrospective research continued	7(32%)

**Table 4:** Types and frequencies of changes made by participating emergency medicine residency programs to resident wellness initiatives during the first New York State wave of the 2019 Novel Coronavirus pandemic.

Change	n(%)
Solicited/obtained donations by outside companies (food, discounts, services)	18(95%)
Scheduled virtual social gatherings	15(79%)
Solicited/obtained donations facilitated/paid for by the hospital	11(58%)
Established new wellness and respite space	9(47%)
Planned for later additional wellness events to make up for canceled plans	8(42%)
Reduced resident workload	8(42%)
Moved residents off high stress/demanding rotations to distribute workflow	7(37%)
Added a virtual class for yoga/meditation	5(26%)

## References

[1] World Health Organization (2020). WHO Director-General’s Opening Remarks at the Media Briefing on COVID-19.

[2] Grissom T, Samet R (2020). The anesthesiologist’s role in teaching airway management to nonanesthesiologists. Adv Anesth.

[3] Ray A, Sole D (2007). Emergency medicine resident involvement in EMS. J Emerg Med.

[4] Vasavda C, Ho B, Davison A (2020). Socially distant medical education in the face of COVID-19. Med Sci Educ.

[5] Emanuel E (2020). The inevitable reimagining of medical education. JAMA.

[6] Wray A, Wolff M, Boysen-Osborn M (2019). Not another boring resident didactic conference. AEM Educ Train.

[7] Hopkins L, Hampton B, Abbott J (2018). To the point: medical education, technology, and the millennial learner. Am J Obstet Gynecol.

