# Supplementary material for: Impact of COVID-19 on Emergency Medicine Residency Programs: A Cross-Sectional Study in New York State
Source: West J Emerg Med. 2022 Jan 18;23(2):246–50. doi: 10.5811/westjem.2021.10.54266 (PMC8967458; doi:10.5811/westjem.2021.10.54266)
Supplement: Supplementary file 1 [file wjem-23-246-s001.docx]

**APPENDIX A**

Electronic Survey Tool Copy

New York American College of Emergency Physicians Residency Program Director Survey:

*Academic Disruption in Emergency Medicine Training Programs During COVID-19 Pandemic*

Select the BEST answer for each of the following questions.

**Residency program:**

- Albany Medical Center
- Arnot Ogden Medical Center
- Brookdale University Hospital & Medical Center
- Good Samaritan Hospital Medical Center
- Icahn School of Medicine at Mount Sinai
- Maimonides Medical Center
- Mount Sinai Morningside
- Nassau University Medical Center
- NewYork-Presbyterian Brooklyn Methodist Hospital
- NewYork-Presbyterian Hospital
- NewYork-Presbyterian Queens
- NYC Health + Hospitals/Coney Island Hospital
- NYC Health + Hospitals/Jacobi
- NYC Health + Hospitals/Lincoln
- NYC Health + Hospitals/Metropolitan
- NYU/Bellevue Emergency Medicine Residency
- Orange Regional Medical Center
- SBH Health System - St. Barnabas Hospital
- Southside Hospital/Northwell Health
- St. John’s Riverside Hospital
- Staten Island University Hospital/Northwell Health
- SUNY at Buffalo
- SUNY at Stony Brook
- SUNY Downstate Medical Center
- SUNY Upstate Medical University
- The Brooklyn Hospital Center
- University of Rochester Medical Center
- Wyckoff Heights Medical Center
- Zucker School of Medicine at Hofstra/Northwell Program at NSLIJ

**Geographic location:**

- Rural
- Suburban
- Urban

**Hospital setting:**

- Academic
- Community
- Government (VA, Military, Etc.)
- Other (please specify)

**Length of Emergency Medicine training program:**

- 3-Year
- 4-Year

**What was the impact of the COVID-19 pandemic on off-service rotations in your EM residency? (Based on the percentage of residents affected).**

- High Impact (61-100%)
- Moderate Impact (41-60%)
- Neutral Impact (21-40%)
- Minimal Impact (< 20%)
- No Impact (no resident affected)

**Did your program cancel any off-service rotations during the COVID-19 pandemic?**

- Yes
- No

**How has the COVID-19 pandemic affected outside rotators in the Emergency Department?**

|  | Cancelled | Postponed | No Change | N/A |
| --- | --- | --- | --- | --- |
| EMT Interns |  |  |  |  |
| Medical Students |  |  |  |  |
| NP Students |  |  |  |  |
| Nursing Students |  |  |  |  |
| PA Students |  |  |  |  |

**Has your program allowed moonlighting for Emergency Medicine residents?**

- Yes
- No
- Unsure

**How did the COVID-19 pandemic impact weekly conferences? (please check all that apply)**

- Canceled
- Changed to Virtual Format
- Limited to Small Groups (< 10)
- Changed to More Self-Directed Learning Activities (i.e. EM:RAP Lectures, ACEP CME, etc.)
- No Change
- Decreased Conference Duration - Please Specify the Number of Hours:

**What changes were made to weekly conferences during the COVID-19 crisis since the national emergency was declared March 13, 2020? (please check all that apply)**

|  | Unchanged | Cancelled | Web-Based Lectures | Other |
| --- | --- | --- | --- | --- |
| Week 1 (3/18/20) |  |  |  |  |
| Week 2 (3/25/20) |  |  |  |  |
| Week 3 (4/1/20) |  |  |  |  |
| Week 4 (4/8/20) |  |  |  |  |
| Week 5 (4/15/20) |  |  |  |  |
| Week 6 (4/22/20) |  |  |  |  |
| Week 7 (4/29/20) |  |  |  |  |
| Week 8 (5/6/20) |  |  |  |  |
| Week 9 (5/13/20) |  |  |  |  |
| Week 10 (5/20/20) |  |  |  |  |
| Week 11 (5/27/20) |  |  |  |  |

**How is the COVID-19 pandemic affecting research in your department? (please check all that apply)**

- Prospective Research Completely Stopped
- Prospective Research Participation Continued Using Video/Phone
- Retrospective Research Stopped
- Retrospective Research Continued
- Other (please specify)

**Has your institution closed or limited the physical presence of non-clinical research staff in the Emergency Department?**

- Yes
- No
- Unsure

**Have you continued to enroll patients prospectively in the Emergency Department?**

- Yes
- No
- Unsure

**Have you made any changes since the start of COVID-19 to assure resident wellness?**

- Yes
- No
- Unsure

**What changes were made since the start of COVID-19 to assure resident wellness? (please check all that apply)**

- Solicited/Obtained Donations by Outside Companies (e.g. Food, Products, Discount, Services, etc.)
- Solicited/Obtained Donations Facilitated/Paid For by the Hospital
- Scheduled Zoom (or Other Virtual) Social Gatherings
- Moved Residents Off of High-Stress/Demanding Shifts/Rotations to Help Distribute the Workflow - Ensured Fair Distribution of Stressful Hours
- Reduced Resident Workload
- Established New Wellness and Respite Space to Provide Emotional and Psychological Support
- Added a Virtual Class for Yoga/Meditation
- Planned for Later Additional Wellness Events to Make Up for Canceled Events During the COVID-19 Shutdown
- Other (please specify):

**Did the Accreditation Council for Graduate Medical Education (ACGME) declare pandemic emergency status for your institution?**

- Yes
- No
- Unsure
